# Establishment and Validation of Reference Genes of *Brassica napus* L. for Digital PCR Detection of Genetically Modified Canola

**DOI:** 10.3390/foods11162535

**Published:** 2022-08-22

**Authors:** Likun Long, Zhenjuan Xing, Yuxuan He, Wei Yan, Congcong Li, Wei Xia, Liming Dong, Ning Zhao, Yue Ma, Yanbo Xie, Na Liu, Feiwu Li

**Affiliations:** Institute of Agricultural Quality Standard and Testing Technology, Jilin Academy of Agricultural Sciences, Changchun 130033, China

**Keywords:** *BnAcc*, *BnC1*, *Brassica napus*, GM canola events, transgenic rapeseed

## Abstract

As an effective tool for genetically modified organism (GMO) quantification in complex matrices, digital PCR (dPCR) has been widely used for the quantification of genetically modified (GM) canola events; however, little is known about the quantification of GM canola events using endogenous reference gene (*ERG*) characteristics by dPCR. To calculate and quantify the content of GM canola using endogenous reference gene (*ERG*) characteristics, the suitability of several ERGs of canola, such as cruciferin A (*CruA*), acetyl-CoA carboxylase (*BnAcc*), phosphoenolpyruvate carboxylase (*PEP*), cruciferin storage (*BnC1*), oleoyl hydrolase (*Fat(A)*), and high-mobility-group protein I/Y (*HMG-I/Y*), was investigated by droplet dPCR. *BnAcc* and *BnC1* were more specific and stable in copy number in the genome of *Brassica napus* L. than the other genes. By performing intra-laboratory validation of the suitability of ERG characteristics for the quantification of GM canola events, the ddPCR methods for *BnAcc* and *BnC1* were comprehensively demonstrated in dPCR assays. The methods could provide technical support for GM labeling regulations.

## 1. Introduction

Canola is widely cultivated in Canada, Europe, and Asia. It is one of the main sources of food and feed, providing edible vegetable oil and protein, and it is the second largest oil crop, accounting for more than 20% of the total edible vegetable oil worldwide, only exceeded by soybean. Genetically modified (GM) canola is one of the four major transgenic crops and is a vital part of genetically modified organism (GMO) safety supervision. As of 2019, more than 40 GM canola events had been authorized as food worldwide [1]. Prior to commercial release, all GM cultivars are required to be assessed for their biosafety to evaluate their potential impacts [2]. The development of transgenic canola quantitative methods provides technical support for the enforcement of regulatory requirements [3,4]. Identification and quantification methods are of great significance not only to ensure legality and traceability but also to comply with GM labeling regulations [5,6]. Generally, the quantitative method for measuring GM content detects the copy number of endogenous and exogenous genes and converts their ratio to actual GM content. Hence, the identification of the appropriate endogenous reference gene (ERG) is the first and most crucial step in the quantification method for each crop. At present, endogenous genes have been developed in many crops, of which maize, soybean, and rice are richest in endogenous genes [3,7,8]. However, the lack of more comprehensive comparative studies on transgenic canola endogenous genes applicable to the digital PCR (dPCR) method renders selection and application for safety supervision difficult [9].

In general, specific and low ERG copies are preferred for the absolute quantification of GM events using PCR [10]. Therefore, only genes with stable low-copy inheritance, the target sequence of which can be efficiently amplified within the minimum partition, are suitable for digital PCR methods. At present, the endogenous genes developed for use in GM canola detection include phosphoenolpyruvate carboxylase (*PEP*) [11], acetyl-CoA carboxylase 8 (*BnAccg8*) [12], cruciferin A *(CruA)* [13], oleoyl hydrolase (*Fat(A)*) [14], cruciferin storage (*BnC1*) [9], and high-mobility-group protein I/Y (*HMG-I/Y*) [15]. Some of these genes have been validated for qualitative detection in transgenic rapeseed, but the quantitative application, especially by digital PCR, requires a clear internal copy number to estimate the content of transgenic components. For example, the uncertainty in the copy number of the commonly used *HMG I/Y* gene as an ERG has become a problem in the quantitative detection of GM in *Brassica napus* L. The *PEP* gene was used for the quantitative and qualitative detection of transgenic *B. napus*, but different amplified target sequences were found [16]. The *CruA* gene was cycled and adopted by the EU transgenic testing laboratory, although Hernandez et al. [12] found that this gene was amplified in other species, and the specificity of *CruA* was low. Therefore, *CruA* is yet to be verified as an ERG using dPCR. Some studies used the *Fat(A)* gene for the identification of GM canola RT73 [17,18,19], but BLASTN results showed that the primer and amplified fragment of *Fat(A)* are homologous to those of that in *Brassica juncea*. Therefore, we evaluated the feasibility of using digital PCR of endogenous genes to satisfy the ERG requirement for GM canola detection [13]. Digital PCR is widely used for the detection or quantification of GM components [20,21]. It can perform absolute quantification and overcome the gaps in real-time PCR, such as PCR amplification efficiency influenced by inhibitors and the bias introduced by the background matrix [22]. In addition, owing to the different amplification principles between dPCR and real-time PCR, the ERGs of real-time PCR may not necessarily be applicable to dPCR [23,24]. Therefore, it is necessary to conduct a comprehensive screening and copy number identification of ERGs in rapeseed and verify the feasibility of dPCR as a method for this purpose [23]. We investigated the equivalence of six validated methods for ERGs and identified the *B. napus*-specific dPCR methods coupled with several event-specific modules for the quantification of specific transgenic canola events.

In this study, the intraspecies conservation and copy numbers of ERGs (*CruA*, *Fat(A)*, *PEP*, *HMG I/Y*, *BnAcc*, and *BnC1*) in common *B. napus* cultivars were assessed using droplet digital PCR (ddPCR). The application of the genes was systematically evaluated. Furthermore, we tested whether *BnAcc* and *BnC1* endogenous genes could be used in quantitative detection of GM content by ddPCR. The methods were assessed by validating the dynamic range, limit of detection (LOD), limit of quantification (LOQ), precision, and applicability. The proposed methods can be applied to the quantitative detection of GM canola content without standard materials. It would provide technical support for labeling regulations of GM canola events and their derived products.

## 2. Materials and Methods

### 2.1. Materials

Seeds of 16 canola cultivars (13 conventional, 3 GM lines) and 8 other crops were acquired for this study. The three GM lines (Ms1, Oxy-235, and Topas 19/2) and one conventional *B. napus* variety were kindly provided by the GM Standard Material Preservation Center (Changchun, China). Twelve other non-transgenic *Brassica* cultivars were provided by Dr. Yuhua Wu of Oil Crops Research Institute of the Chinese Academy of Agricultural Sciences, including three cultivars of *B. juncea* (‘Simianhanzha’, ‘AB1’, and ‘Lu’), three cultivars of *B. rapa* L. (‘Shanghaiqing’, ‘10wH008’, and ‘Huangxinwu’), four cultivars of *B. napus* (‘AV-jade’, ‘Zhongshuang-11’, ‘Zhongshuang-b’, and ‘Nh No.345’), and one cultivar each for *B. oleracea* L. (‘Niuxin’), *B. nigra* (L.) W.D.J.Koch (‘black mustard’), and *B. carinata* A. Braun (‘Ethiopian mustard’). The seeds of the eight other crops (*Solanum tuberosum* L., *Lycopericon esculentum* Mill., *Nicotiana tabacum* L., *Raphanus sativus* L., *Oryza sativa* L., *Glycine max* (L.) Merr., *Zea mays* L., and *Gossypium hirsutum* L.) were purchased from a local market. Samples were collected and stored in the laboratory at −20 °C.

### 2.2. Preparation of Samples with Different GMO Contents

All acquired seeds were crushed by a mixer (Retsch MM430, Shanghai Vedder Instrument Equipment Co., Ltd. Shanghai, China). The grinding process involved ten cycles of 20 s agitation, followed by cooling to room temperature (20–25 °C) for 1 min to protect the DNA from heat damage. After grinding, 10%, 1%, 0.5%, and 0.1% MS1, Oxy-235, and Topas 19/2 transgenic samples (by mass ratio) were prepared according to the material preparation process (Announcement No. 1782 of the Ministry of Agriculture of China, Technical Specification for Preparation of Transgenic Standard Materials). The powder was further mixed in a Dynamic CM-200 mixer (Retsch, Germany) and shaken overnight to obtain homogenous samples for DNA extraction.

### 2.3. DNA Extraction

DNA was extracted from 50 mg of seed powder using a DNeasy Plant Mini Kit (Qiagen, Hilden, Germany) following the manufacturer’s guidelines. DNA concentration and purity were determined using a NanoDrop N2000 instrument (Thermo Fisher Scientific, Wilmington, DE, USA). Samples were diluted to a final concentration of 12.5 ng/μL.

### 2.4. Primers and Probes

The primers and probes of six sets of endogenous and three sets of transgenic event-specific genes were used in our methods (Table 1). The oligos were synthesized by Sangon Biotech (Shanghai, China).

### 2.5. Real-Time PCR

Real-time PCR was used to analyze the copy numbers of endogenous genes and transformant-specific genes by the standard curve method [28].

The 1× HiTaq probe Mastermix (Apexbio Biotechnology, Beijing, China), 500 nM of each primer, and 250 nM of target and reference gene probes were included in 25-μL qPCR reactions. PCR amplification was performed on a CFX96 real-time thermal analyzer (Bio-Rad, Hercules, CA, USA). Unless specified otherwise, the template was 12.5 ng of genomic DNA. FAM (6-carboxyfluorescein) channels for ERGs and GM-specific fragments were amplified following the thermal cycle protocol: 95 °C for 5 min, 35 cycles at 95 °C for 15 s, and 60 °C for 1 min. Fluorescence signals were read out during the extension steps. All reaction data were analyzed using Opticon Monitor_2 software version 2.02 (MJ Research, Waltham, MA, USA). PCR reactions were conducted in three parallel assays.

### 2.6. Droplet Digital PCR

A droplet digital PCR system (QX200, Bio-Rad) was used for the ddPCR method [23]. Each 20-μL reaction mixture consisted of 500 nM of the forward and reverse primers, 250 nM of the target and reference gene probes, 10 μL of ddPCR Supermix (L002054A, Bio-Rad), and 12.5 ng of DNA template. According to the manufacturer’s instructions, 20 μL of ddPCR reaction mixture and 70 μL of droplet formation oil (EvaGreen 186-3005, Bio-Rad) were placed in the cartridges, and droplets were generated on the AutoDG instrument (Bio-Rad). Then, the nanoliter droplets were dispensed into a 96-well dPCR plate (1200–1925, Bio-Rad) to perform the amplification in an AC1000 PCR Thermocycler (Bio-Rad). The PCR reactions were carried out under the following conditions: 10 min at 95 °C for denaturation, 40 cycles at 95 °C for 30 s, 58.5 °C for 60 s, and 98 °C for 10 min. The plate in which PCR was completed was put into a QX200 instrument for signal readout and data analysis. For each experiment, three duplicate samples were analyzed, unless otherwise noted.

Thresholds were manually set based on fluorescence amplitudes and numbers of events (1D amplitudes) on the FAM channel, and histograms of event and amplitude data streams were used to distinguish the positive droplets from negative droplets without amplification products. If clog was detected by the software (Bio-Rad QuantaSoft version 1.6.6) or the number of droplets detected per 20 μL of reaction was low (<8000), the data were rejected for subsequent analysis [22].

### 2.7. LOD and LOQ of Digital PCR Methods

gDNA was extracted from the seeds of GM canola Ms1, Oxy-235, and Topas 19/2. A diluted series copy number sample for the target copy was prepared, and the theoretical absolute copy number was converted from each concentration of diluted DNA samples according to the haploid genome of 1.3 pg [29]. The absolute limit of quantification (aLOQ) in copy number and absolute limit of detection (aLOD) in copy number were determined from the results of 15 replicates by ddPCR. aLOD is the lowest copy sample consistently producing a positive signal in all replicates, whereas aLOQ is the lowest copy tested with a relative standard deviation (RSD) of <25% [11].

### 2.8. Determination of the Dynamic Range

The logarithm of experimental (ddPCR) and theoretical (gDNA) copies of *B. napus* was used to generate a linear regression curve. The regression coefficient (R^2^) was used to evaluate the dynamic range of the ERGs quantitative method [30]. In this experiment, repeatability was evaluated through a series of diluted DNA samples (two separate runs; two replicates were performed, on day 1 and day 2).

### 2.9. Method Verification

Methods were validated according to a document [31]. The criteria of the dynamic range, trueness, and precision were verified by the mixed gDNA samples with different mass ratios (10%, 5%, 1%, and 0.1% of MS1, Oxy-235, and Topas 19/2) by real-time PCR and ddPCR. The assays of two endogenous genes (*BnAcc* and *BnC1*) and the GM-specific genes of MS1, Oxy-235, and Topas 19/2 events were used to quantify the practical content of the GM. Furthermore, the absolute copy numbers of the specific and endogenous genes were calculated using the Opticon Monitor_2 software version 2.02 for real-time PCR and QuantaSoft software version 1.6.6 (Bio-Rad) for ddPCR. The GMO content was calculated as the ratio of copy numbers of the specific gene to the endogenous gene. The data measured were compared with the mass ratio of the sample standard prepared.

### 2.10. Data Analysis

Real-time PCR data were processed using Bio-Rad Opticon Monitor_2 software version 2.02. All biological and technical replicates were used to calculate the average Ct value. The copy number of the targets (exogenous or endogenous gene) was calculated using the standard curve according to the equation x = 10^[(Ct − b)/a]. ddPCR data were analyzed using QuantaSoft software version 1.6.6 (Bio-Rad Laboratories). The GM content was calculated as (target DNA copy number)/(reference DNA copy number) × 100 [32].

## 3. Results and Discussion

### 3.1. Evaluating Intraspecies Inclusiveness and Species Specificity of Canola

Intraspecific inclusion and species specificity are two basic requirements for reference gene selection. Six primers/probe sets (Table 1) were used for the real-time fluorescence quantitative PCR detection of six ERGs. The samples were all from different crop species and cultivated varieties of *Brassica L*. to verify whether there was a cross-reaction of endogenous genes or non-targeted specific genes of other genera. 

The samples used in the study included cultivars of six *Brassica* species: *B. rapa*, *B.* *nigra*, *B. oleracea*, *B. juncea*, *B. napus*, and *B. carinata*. All six endogenous genes had no homologous sequences in maize, soybean, rice, and other crops but had positive amplified fragments in all *B. rapa*, *B. juncea*, and *B. napus* varieties (Table 2), suggesting that these six genes do not distinguish *B. napus* from other cultivars. As most GM canola events were in *B. napus*, our goal was to screen out the specific ERG of *B. napus*. However, none of these six PCR systems exhibited *B. napus* specificity. This result was consistent with a previous report [13].

*PEP*, *CruA*, and *Fat(A)* showed similar amplification specificity in the test crops. Their fragments could be identified not only from the genomes of all six *Brassica* species but also from *R. sativus*. In the *HMG-I/Y* PCR reaction, the fragments could be amplified in *B. rapa*, *B. juncea*, *B. napus*, *B. oleracea*, and *B. carinata.* The *BnAcc* system exhibited a performance similar to that of *BnC1* in the tested cultivars of *B. rapa*, *B. juncea*, and *B. napus*. Aside from the positive result of *BnAcc* in *R. sativus*, ERGs of *BnAcc* and *BnC1* fragments were restricted to the *B. rapa*, *B. juncea*, and *B. napus* cultivars (Table 2). 

All amplified fragments from different cultivars of these six genes were sequenced and used for sequence comparison. Apart from some single nucleotide polymorphisms, no large interspecific variation was detected in the amplified fragments. 

In summary, the six endogenous genes could be used to distinguish canola from other crops, but none was specific to canola varieties. Although the specificity was different, PCR confirmed the presence and consistency of the genes in *B. napus*. *BnAcc* and *BnC1* genes exhibited a higher specificity than the others. Their amplification was specific in *B. rapa*, *B. juncea*, and *B. napus*, but not in *B. nigra*, *B. carinata*, *B. oleracea*, and other crop varieties, unlike the other ERGs. As the current transgenic rapeseed varieties mainly belong to *B. napus*, these endogenous genes are probably applicable to quantitative detection as reference genes for *B. napus*. Therefore, the stability of the copy number of these six genes in *B. napus* was the main basis for further selection.

### 3.2. Verifying the Stability of Copy Numbers of ERGs among B. napus Cultivars by Droplet dPCR

The copy number stability among diverse cultivars is a vital factor in developing ERG assays for GM detection. To identify the stability of the ERGs within *B. napus* cultivars, the homogeneity of the genes in different genomes needs to be considered. In accordance with EU requirements, ERGs and corresponding quantitative methods must be stable in different *B. napus* varieties. To evaluate the potential variation of these genes, gDNA of seven *B. napus* cultivars, including three GM and four non-GM canola, was analyzed by quantitative ddPCR systems for six ERGs: *CruA*, *Fat(A)*, *PEP, HMG I/Y*, *BnAcc,* and *BnC1* (Table 3).

Each sample was used as a PCR template for six endogenous reference systems, and the amplification was repeated twice for two runs. After the reactions, the copy number of the ERGs in cultivars was detected. The mean copy number of each gene was calculated as shown in Table 3. Thus, the stability of these six endogenous reference systems was compared and analyzed by standard deviation (SD) and relative standard deviation (RSD). The number of *HMG-I/Y*, *Fat(A)*, and *CruA* exhibited greater variability than that of the other genes in *B. napus* cultivars, with an RSD of 7.90%, 7.21%, and 9.18%, respectively (Table 3).

**Figure 1:**
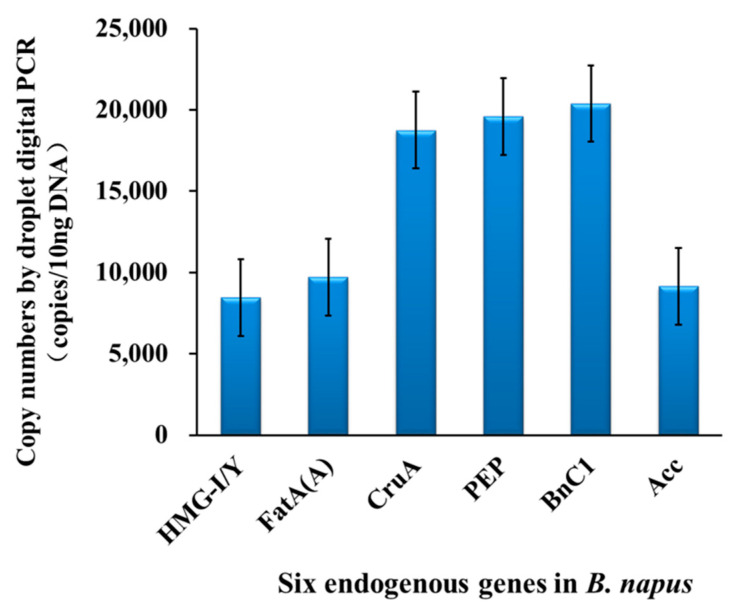
Average copy numbers for six ERGs (average of seven *B. napus* cultivars) plus standard deviation. For ddPCR, 12.5 ng DNA was used. The experimental data include all cultivars of *Brassica napus*, including GM canola of MS1, Oxy-235, and Topas 19/2 and four non-GM canola of AV-Jade, Zhongshuang-11, Nh No.345, and Zhongshuang–b.

The copy number of *CruA*, *PEP*, and *BnC1* was double that of *HMG-I/Y*, *Fat(A),* and *BnAcc* using the same samples and ddPCR reactions (Figure 1). The copy number of *hmg-I/Y*, *Fat(A)*, and *BnAcc* measured by dPCR was close to the theoretical copy number of a single copy in the *B. napus* genome, which was consistent with the results of previous studies [11,12,17]. The quantitative results of six dPCR methods were close to the theoretical values of single or double ERG copies in the *B. napus* genome, with deviations of less than 5%. 

### 3.3. Assessment of ddPCR for ERGs

#### 3.3.1. Selection from Observations of ddPCR

**Figure 2:**
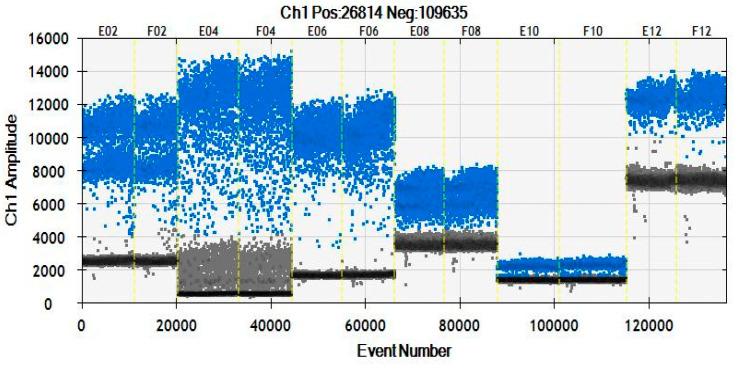
ddPCR amplitude plots for *Brassica napus* cultivar of six reference genes. Amplification of *CruA*, *HMG*, *BnC1*, *PEP*, *Fat(A)*, *BnAcc* genes from left to right. Blue, positive droplets; black, negative droplets. E02 and E04 to F12 are the detection positions in the 96-well PCR plate by QX2000.

The observations of ddPCR amplitude plots for the six ERGs are shown in Figure 2. From the distribution of the droplets, the threshold limits of negative and positive droplets were not obvious for the *Fat(A)* assay. Setting the threshold manually may affect the accuracy of a low copy number determination. For *CruA* and *HMG I/Y* assays, there were some droplets in the amplification plot that interfered with the accuracy of the quantitative detection, whereas there was a clear separation of positive and negative clusters in the *BnC1* and *BnAcc* gene amplification. Therefore, the analysis of copy number stability and amplification showed that *BnC1* and *BnAcc* were accurate and reliable. They could be candidate ERG assays for the quantitative detection of GM canola using ddPCR.

#### 3.3.2. aLOQ and aLOD of ddPCR of BnC1 and BnAcc

aLOD can be defined as the minimum amount of analyte in a test sample that can be detected reliably [33], whereas aLOQ is the lowest amount of analyte that can be determined quantitatively with accuracy and precision [31]. In the dPCR, the lowest copy number at which all replicates were detected positively was set as the aLOD. gDNA samples containing 20, 10, 5, 2, and 1 ERG copies were prepared from the Ms1 DNA, with the purpose of determining the aLOD and aLOQ. They were measured using ddPCR assays at five levels of dilution, with 15 PCR replicates targeting these ERGs. From the positive rate of amplification, all digital PCR replicates were 100% amplified in more than 10 copies (Table 4). In five copies, the positive rate of *CruA*, *BnC1*, and *BnAcc* was 100%; thus, these three ERGs have a lower LOD with about five copies. The LOD of *Fat(A)* and *PEP* was higher than that of the above three genes, with 10 copies. RSD analysis was performed on these low copy samples using copy number values obtained in the digital PCR. The RSD increased, whereas the copy number decreased. Thus, aLOQ can be estimated as the lowest copy where the RSD of the measurements is below 25%. When the copy number was above ten, the RSD values of each ERG were below the criteria, whereas in five copies for sample amplification, *BnC1* and *BnAcc* exhibited 24.8% and 22.5%, respectively. That means the aLOQ of *BnC1* and *BnAcc* could reach five copies. Considering the LOQ was for the ERGs instead of specific genes, this was a satisfactory result, as the content of the exogenous targets was not less than that of the ERGs in the GM detection. Therefore, *BnC1* and *BnAcc* have stronger specificity in *B. napus* and higher sensitivity than the other four genes as ERGs for GM canola detection.

#### 3.3.3. Dynamic Range

**Figure 3:**
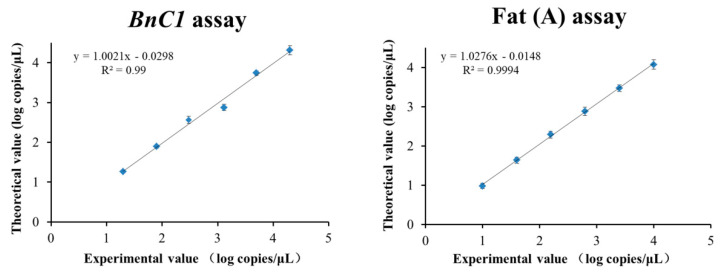
Dynamic range and correlation between experimental copy numbers with assigned copy numbers of *BnC1* and *BnAcc* assays. The average data of three independent experiments are represented (R^2^ values for *BnC1* and *BnAcc* are 0.99 and 0.9994, respectively).

For the specific *B. napus* endogenous genes of *BnAcc* and *BnC1*, the linear relationship between the theoretical and actual copy numbers of both reference genes was evaluated using ddPCR. Rapeseed DNA samples were diluted to different concentrations in the absolute amount in the reaction system, with 10,000, 2500, 625, 156, 40, and 10 copies. The theoretical copy number of *BnC1* was double that of *BnAcc* in the rapeseed genome. The R^2^ coefficient represents a measure of the estimative capacity of the linear regression line in relation to the experimental data used. The result indicated that the copy numbers of both genes were linear along the entire copy range (10,000 to 10 copies of *BnAcc*; 20,000 to 20 copies of *BnC1*), with R^2^ > 0.99 (Figure 3). Therefore, the linear range of the two methods can fully meet the required detection needs and can be considered as equivalent. As the content of endogenous genes in transgenic samples is often equal to or higher than the content of specific genes, this indicated that the ratio of specific genes to sample ERGs is sufficient to ensure detection. The achieved dynamic range allows for the characterization of GM rapeseed, while covering almost the entire specific concentration range that is possible in a GM sample, or the most probable concentrations.

#### 3.3.4. Quantitative Detection of GM Canola Events by Real-Time PCR and ddPCR with BnC1 and BnAcc

**Figure 4:**
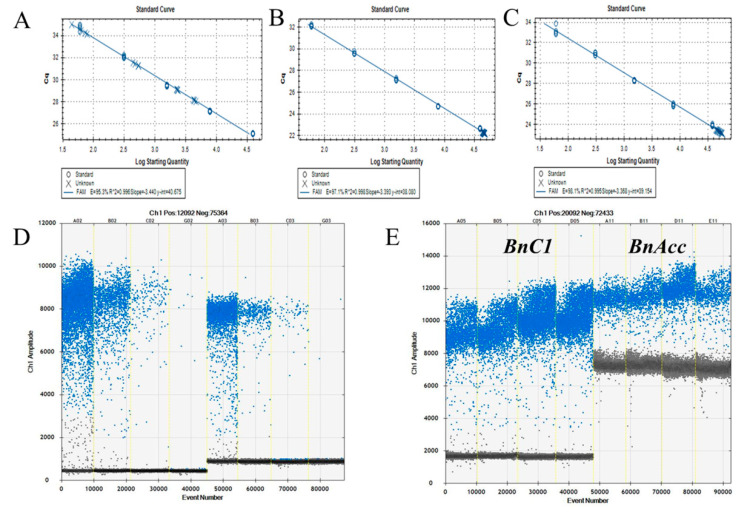
Validation of the RT-PCR and ddPCR methods using blinded samples with ERGs of BnAcc and BnC1 assays. The standard curves for the MS1-specific RT-PCR method using gradient-diluted MS1 genomic DNA as the template were analyzed using CFX96 System. gDNA samples with mass fractions of 10%, 5%, 1%, and 0.1% of MS1 were used as a template to quantify the specific MS1 gene (**A**) and reference *BnC1* (**B**) and *BnAcc* (**C**) on the RT-PCR platform. The same samples were amplified on a ddPCR of MS1-specific gene (**D**); amplification of *BnC1* and *BnAcc* genes (**E**).

To ascertain whether the ERG methods of dPCR could achieve accurate results for the practical quantification of the GM canola content, we conducted endogenous gene assays using test samples that were mixed by mass ratio. The GM contents were prepared for 10%, 5%, 1.0%, and 0.1% of MS1, Oxy-235, and Topas 19/2. For comparison, both real-time PCR and ddPCR were conducted on these samples, with event-specific genes and both *BnC1* and *BnAcc* as ERGs. The copy number by real-time PCR was calculated using the standard curve established by reference gDNA dilutions for the MS1 gene, *BnC1*, and *BnAcc* (Figure 4A–C). Droplet digital PCR was directly used to quantify the positive droplets of the amplification of exogenous genes (Ms1 and Oxy235 as examples, Figure 4D) and endogenous genes (*BnC1* and *BnAcc*) (Figure 4E). We assessed 10%, 5%, 1%, and 0.1% of MS1, Oxy-235, and Topas 19/2 rapeseed DNA samples using two ERGs by dPCR and real-time PCR for the quantification. The comparison and analysis of the quantitative determination results and theoretical content by the two platforms for 12 blind samples are shown in Table 5. 

The dPCR amplification plot indicated that the assays could achieve the expected result with the endogenous genes (*BnC1* or *BnAcc)*. The error indicated as bias (%) of all quantitative values was within ±25% of the European Network of Transgenic Laboratories recommended values [31]. The maximum error bias of dPCR in the result was −22.24% for the Topas 19/2 samples, with 0.1% content determined by *BnAcc* as the ERG, whereas the maximum error bias of real-time PCR was 22.17%, as for Topas 19/2 samples, with 0.1% content determined by *BnC1* as the ERG. Both RT-PCR and dPCR methods can achieve accurate results for both ERGs in transgenic samples with content as low as 0.1%. The results indicated that either the *BnC1* or *BnAcc* gene was appropriate for the quantitative assessment of GM canola events. Moreover, real-time PCR and ddPCR methods could achieve accurate and consistent results. Compared with those of reported methods [4,14,17], the *BnC1* and *BnAcc* assays used in ddPCR assays achieved satisfactory results in GM canola quantification. The single or double copies for reference genes did not affect the ddPCR evaluation of the GM content in these three GM canola events in *B. napus*. The applicability of dPCR targeting *BnC1* and *BnAcc* genes was successfully demonstrated. Moreover, the dPCR methods using *BnC1* and *BnAcc* as reference genes were suitable for the quantitative detection of transgenic rapeseed. 

In some studies of qualitative GM detection methods in canola, some EGRs were used for the identification and quality control of extracted canola genomic DNA. *CruA* and *HMG I/Y* are reference genes used by some researchers for quantitative detection [7,28,34]; however, the specificity and copy number stability of all reference genes in *B. napus* have not been systematically studied. Wu et al. [13] used reference genes for GM content detection in real-time PCR, but real-time PCR detection depends on reference material, and plasmids and the homozygous genome as reference material were always unavailable. The superior characteristics of *BnC1* and *BnAcc* determined in our study were demonstrated to be suitable for quantitative detection by digital PCR. With the advantages of the same accuracy as real-time PCR and independence of standard materials in the process of detection, the dPCR platform would be more convenient for practical application to quantify the content of GM canola and their derived products compared to real-time PCR or the other routine techniques.

## 4. Conclusions

The criteria of six canola genes (*CruA*, *BnC1*, *Fat(A)*, *PEP*, *HMG I/Y*, and *BnAcc*) were compared and evaluated using ddPCR in this research. With their combined specificity, stability, dynamic ranges, and amplification observation, both *BnC1* and *BnAcc* could be used as ERGs for the quantification of GM canola content in ddPCR assays. Using the ERGs, a ddPCR system was established with an aLOQ of 10 copies/reaction. This method can reliably quantify the GM content, even when it is as low as 0.1%. Different content of MS1, Topas 19/2, and Oxy235 GM canola events validated that *BnC1* and *BnAcc* endogenous genes were reliable in both real-time PCR and ddPCR with the same primers and probes. Concluding from these results, we believe that this newly developed method will be a good candidate of using ERGs for quantifying the amount of GM canola by dPCR. These methods can provide reliable and reproductive results and perform as effective tools to implement labeling systems.

## Figures and Tables

**Table 1:** Primers and fluorescence probe used in this study.

Gene	Accession No.	Primer/Probe Name	Sequences 5′-3′	Amplicon (bp)	Resource
*CruA*	X14555	CruA-F	GGCCAGGGTTTC CGTGAT	101	[13]
		CruA-R	CCGTCGTTGTAGAACCATTGG		
		CruA-P	FAM-AGTCCTTATGTGCTCCACTTTCTGGTGCA-TAMRA		
*Fat(A)*	AJ294419	Fat(A)-F	ACAGATGAAGTTCGGGACGAGTAC	126	[7]
		Fat(A)-R	CAGGTTGAGATCCACATGCTTAAATAT		
		Fat(A)-P	FAM-AAGAAGAATCATCATGCTTC-TAMRA		
*HMG-I/Y*	AF127919	HMG-F	GGTCGTCCTCCTAAGGCGAAAG	99	[15]
		HMG-R	CTTCTTCGGCGGTCGTCCAC		
		HMG-P	FAM-CGGAGCCACTCGGTGCCGCAACTT-TAMRA		
*BnC1*	X59294.1	Ccf-F	ATTGGGCTACACCGGGATGTGT	96	[18]
		Ccf-R	GCTTCCGTGATATGCACC AGAAAG		
		Ccf-P	FAM-CGATGGTGTCCCCAGTCCTTATGTGCTC-TAMRA		
*PEP*	D13987	pep-F	CAGTTCTTGGAGCCGCTTGAG	140	[12]
		pep-R	TGACGGATGTCGAGCTTCACA		
		pep-P	FAM-ACAGACCTACAGCCGATGGAAGCCTGC-TAMRA		
*BnACC*	X77576	Acc-F	GGTGAGCTGTATAATCGAGCGA	104	[11]
		Acc-R	GGCGCAGCATCGGCT		
		Acc-P	FAM-AACACCTATTAGACATTCGTTCCATTGGTCGA-TAMRA		
*MS1*	EU090198	Ms1-F	ACGCTGCGGACATCTACATT	187	[25]
		Ms1-R	CTAGATCGGAAGCTGAAGATGG		
		Ms1-P	FAM-CTCATTGCTGATCCACCTAGCCGACTT-TAMRA		
*OXY235*	KJ608141	Oxy-F	ATTGACCATCATACTCATTGCTGA	105	[26]
		Oxy-R	AGAGAATCGTGAAATTATCTCTACCG		
		Oxy-P	FAM-CCATGTAGATTTCCCGGACATGAAGCC-TAMRA		
*Topas 19/2*	EU124676	Topas-F	GTTGCGGTTCTGTCAGTTCC	95	[27]
		Topas-R	CGACCGGCGCTGATATATGA		
		Topas-P	FAM-TCCCGCGTCATCGGCGG-TAMRA		

**Table 2:** Specificity analysis of the endogenous reference genes.

Species/Cultivars	Sample Name	*CruA*	*Fat(A)*	*HMG-I/Y*	*BnC1*	*PEP*	*BnAcc*
*Brassica juncea*	Lu	+	+	+	+	+	+
	Simian Shanzha	+	+	+	+	+	+
	AB1	+	+	+	+	+	+
*Brassica rapa*	Shanghai Qing	+	+	+	+	+	+
	Huangxin Wu	+	+	+	+	+	+
	10 wH008	+	+	+	+	+	+
*Brassica napus*	AV-Jade	+	+	+	+	+	+
	Zhongshuang-11	+	+	+	+	+	+
	Nh No.345	+	+	+	+	+	+
	Zhongshuang-b	+	+	+	+	+	+
	Ms1	+	+	+	+	+	+
	Oxy-235	+	+	+	+	+	+
	Topas 19/2	+	+	+	+	+	+
*Brassica oleracea*	Chinese kale	+	+	+	-	+	-
*Brassica nigra*	black mustard	+	+	-	-	+	-
*Brassica carinat*	Ethiopian mustard	+	+	+	-	+	-
*Raphanus sativus* L.	radish	+	+	-	-	+	+
*Nicotiana tabacum*	tobacco	-	-	-	-	-	-
*Solanum tuberosum*	potato	-	-	-	-	-	-
*Lycopericon esculentum*	tomato	-	-	-	-	-	-
*Glycine max*	GM Soybean	-	-	-	-	-	-
*Zea mays*	GM corn	-	-	-	-	-	-
*Oryza sativa*	GM rice	-	-	-	-	-	-
*Gossypium hirsutum*	GM cotton	-	-	-	-	-	-

Real-time PCR of these six ERG methods was performed in this test. (-) Negative result. (+) Positive result.

**Table 3:** Copy number variation of reference targets among seven *Brassica napus* cultivars.

Endogenous Genes	Copy Numbers in Cultivars of *Brassica napus.* (12.5 ng Genome DNA)							Average	RSDr ^a^
	MS1	Oxy-235	Topas 19/2	AV-Jade	Zhongshuang-11	Nh No.345	Zhongshuang-b		
*HMG-I/Y*	9049	7956	8956	7463	8856	9020	7856	8451	7.90
*Fat(A)*	9696	9731	9719	9688	10,210	8060	9860	9709	7.21
*CruA*	20,235	17,153	19,560	15,800	18,963	18,930	20,650	18,756	9.18
*PEP*	19,242	19,834	20,157	20,688	20,260	16,510	19,560	19,607	7.13
*BnC1*	20,643	20,501	20,189	20,438	21,000	21,300	18,693	20,538	4.10
*BnAcc*	9445	8862	9216	8675	8657	9560	9650	9152	4.59

^a^ RSDr means the relative standard deviations of the average copy number of seven *B. napus* cultivars.

**Table 4:** aLOD and aLOQ of these six ERGs assays of *Brassica napus.*

Target	Template Copy No.	Signal Ratio	Mean Copy No.	RSD Copy No. (%)
*HMG-I/Y*	20	15/15	16	14.9
	10	15/15	8	23.5
	5	15/15	6	41.4
	2	7/15	/	/
	1	5/15	/	/
*Fat(A)*	20	15/15	19	12.2
	10	15/15	9	22.6
	5	12/15	/	/
	2	10/15	/	/
	1	4/15	/	/
*CruA*	20	15/15	22	19.8
	10	15/15	13	24.7
	5	15/15	8	45.8
	2	10/15	/	/
	1	5/15	/	/
*PEP*	20	15/15	23	13.5
	10	15/15	13	20.9
	5	13/15	/	/
	2	7/15	/	/
	1	5/15	/	/
*BnC1*	20	15/15	21	12.1
	10	15/15	11	20.3
	5	15/15	8	24.8
	2	11/15	/	/
	1	8/18	/	/
*BnAcc*	20	15/15	22	14.7
	10	15/15	10	18.9
	5	15/15	6	22.5
	2	8/15	/	/
	1	6/15	/	/

“/” means no data be calculated.

**Table 5:** Droplet digital PCR results obtained for GE canola samples using six reference genes.

GM Rapeseed Event	Method	Exogenous Gene		*BnAcc*			*BnC1*		
		GM Content by Mass Ratio (%)	Average Copy Numbers	Average Copy Numbers	Experimental GM Concentration (%)	Bias %	Average Copy Numbers	Experimental GM Concentration (%)	Bias %
MS1	Real-timePCR	10	1056	9830	10.74	7.43	20,715	10.20	1.96
		5	551	10,840	5.08	1.66	21,385	5.15	3.06
		1	110	9687	1.14	13.55	20,380	1.08	7.95
		0.1	13	9937	0.12	20.76	20,150	0.11	19.11
	ddPCR	10	890	9600	9.27	−7.29	20,925	8.51	−14.93
		5	517	9490	5.45	8.96	20,824	4.97	−0.69
		1	102	9138	1.12	11.62	20,610	0.99	−1.02
		0.1	9	9088	0.10	−0.97	20,700	0.09	−13.04
Topas 19/2	Real-timePCR	10	1100	10,863	10.13	1.26	20,438	10.76	7.64
		5	540	9445	5.72	14.35	20,697	5.22	4.36
		1	96	9160	1.05	4.80	21,104	0.91	−9.02
		0.1	11	9100	0.12	20.88	18,008	0.12	22.17
	ddPCR	10	952	10,800	8.81	−11.85	19,938	8.15	−18.55
		5	406	9507	4.27	−14.59	20,680	5.67	13.35
		1	85	10,563	0.80	−19.53	21,104	0.81	−19.45
		0.1	8	10,288	0.08	−22.24	19,038	0.08	−15.96
Oxy235	Real-timePCR	10	1101	10,925	10.08	0.78	19,938	11.04	10.44
		5	499	9407	5.30	6.09	18,963	5.26	5.26
		1	110	10,563	1.04	4.14	20,650	1.07	6.54
		0.1	11	9289	0.12	18.42	18,130	0.12	21.35
	ddPCR	10	966	10,920	8.85	−11.54	17,360	11.13	11.29
		5	478	8657	5.52	10.43	19,070	5.01	0.26
		1	100	9240	1.08	8.23	19,830	1.01	0.86
		0.1	9	9650	0.09	−6.74	21,038	0.09	−14.44

## Data Availability

The data are available from the corresponding author.
